# The Neglect of the Gold Foil Filling

**Published:** 1913-03-15

**Authors:** Charles C. Johnson

**Affiliations:** Jackson, Mich.


### THE NEGLECT OF THE GOLD FOIL FILLING.

BY CHARLES C. JOHNSON, D.D.S., JACKSON, MICH.

In selecting a subject for discussion at this meeting it might appear less presumptious or at least more appropriate for a young man to choose a subject along the lines of the more popular methods; but, since most of the newer subjects especially those which I would care to write about have been thoroughly discussed in this society recently; perhaps it will not seem imprudent on my part when I attempt to express my ideas on a subject which is so universally practical that it ought to bring out a good discussion, which perhaps may be far better than a dozen papers written on the subject. Hence the title selected for the theme of the paper and with which the writer will endeavor to introduce his ideas in as few words as possible, will be: "The Neglect of the Gold Foil Filling."

In this day and age of modern dentistry we find there is a much larger field for us to work than there was only a few years ago. In our present field we have specialists in several different lines: as the orthodontist, the anesthetist and the oral surgeon. Some are very skilled workmen along certain lines, as in the manipulation of gold for crowns, bridges, inlays and gold fillings. Again we have some who may not be especially skilled technically, that is, they may not possess unusual mechanical ability, but who are great writers or research workers along certain lines and then we have some who are devoting time and energy to teaching the relationship of the teeth to the general health of the body. We are fortunate in possessing a field capable of attracting so many skilled workers and are glad to recognize them and to give them their respective places in modern dentistry.

From a study of the present conditions, and as we look into the future of dental practice we can see that we are approaching a crisis in the profession along some lines of practice. If you will pause to think, you will agree with me in this matter, for change must come with the passing years, and just as each generation must alter conditions to meet the needs of its time, so must we alter our methods to meet the new demands made necessary by changes of society and the individual.

As has been said, dentistry as a calling has made marvelous progress in the last few years and had we the time it would be interesting and profitable to trace this progress in all branches, for it is well worthy of our consideration. Perhaps the subject which has been creating the most discussion lately is oral hygiene. It is not only receiving serious consideration among the practitioners of our own profession, but it is attracting the attention of the medical profession and educated people of all civilized countries. And so we find as practitioners many pathways to be pursued in our special field; but before we can obtain the more lucrative practice or attain the higher positions in the profession, we must know first how to accomplish the more simple operations or usual methods of practice known to dental surgery.

We find the majority of dentists, at least the average practitioners, are to deal with the restoration to the normal of lost tooth structure caused by dental caries. Not drifting from the subject further, let us take up the more practical or common ways of restoring lost tooth structure to as near a normal condition as may be practicable. Prior to the year 1907, we find we have for the selection of filling materials, the porcelain inlay, the gold filling, amalgam and oxyphosphate cements; then comes Dr. Taggart, the dental wizard, who tells us how to make a mould from a wax pattern and therefrom obtain an exact form in gold, by the casting process. We might truthfully say that gold casting alone has completely revolutionized modern dentistry; it is certainly one great stride in the development and progress of the profession in general.

Be the *cast gold process* what it may, especially the cast gold inlay for restoring lost tooth structure, it should never, in the writer's estimation, intrude or supplant the one old standby which has stood the test of time, which is with us now and always will be, namely: The Gold Foil Filling.

We should say that all the other materials used in their various forms for filling teeth have their respective places at the option or discretion of the operator. The *basis* for the *selection* and *adaptation* of the various classes of cases for filling materials lies within the individual operators own judgment as to which he can best manipulate so as to make the most permanent filling, considering the case, his own skill, and the durability of the material.

I believe the basis for selecting a filling material is generally controlled, at least in the majority of cases, by one's ability to manipulate the different materials successfully or easily. In many cases dentists use what may be manipulated for the ease of their patient or for their own convenience, and instead of forming a perfect cavity and putting in gold filling with the proper density and polish, they will slice off the tooth and place either a gold inlay or an unreliable amalgam filling. We will all concede that all the filling materials used in dentistry have their respective places under *certain* conditions, are ideal both to patient and operator, such as the porcelain inlay, the gold inlay, the gold filling, silicate cements, and amalgam filling. Not for a minute would the writer question the durability and the beautiful results of our good porcelain workers, because the gold foil filling would never be *abused* or *neglected* by the artistic use of this material. Neither would he question the selection and adaptation of the gold inlay when a proper case *presents* itself and the *technique* is ideal from a practical standpoint; but in many cases we have questioned not only the poor technique and the bad judgment of the operator in choosing the inlay to the neglect of the Gold Foil Filling, which was indicated not only from the technical but the practical point of view. It may seem presumptuous for me to seem to instruct in the art of filling a tooth with gold foil, as the subject is so trite that it would seem there was nothing to be said on so fundamental a subject. But in our anxiety to learn to use the newer methods I think I notice a tendency to neglect some of the fundamental principles involved in producing good gold operations. We should establish asepsis and maintain the same throughout the entire operation. I think some of us are getting careless in regard to this important matter. Proper cavity preparation perhaps is one of the greatest stumbling blocks and in the main is the cause for so many failures particularly in Gold Foil Fillings as well as in all other fillings. One of the noticeable faults in cavity preparation for anterior fillings is that we do not sacrifice enough tooth structure on the lingual wall, thereby inviting a leaky joint and discoloration, with an ultimate general breaking down. We would suggest cutting the lingual wall away liberally. The annealing of the gold and its inserting into the cavity is largely a matter of personal preference; but one thing we must studiously avoid, and that is all possible contamination which is a great injury to Gold Filling. Proper density and specific gravity. Perhaps one of our greatest troubles is that we do not get the proper density; edges are poorly condensed which causes imperfect margins, resulting in a leaky filling. The specific gravity of the cast gold filling is about nineteen. Average operators get about twelve to fifteen condensings with the mallet. And last but not least, is the finish of the filling. Many a fair filling is put in only to give way from the lack of well burnished edges and a good polish on all surfaces. It must be said that we are all inclined to take life easy, but if more pains were taken to burnish and polish the distal and lingual surfaces of gold fillings, even as well as we do the labial surfaces, many of us would have a clearer conscience and enjoy the practice of our old patients rather than having to solicit new ones all the time.

I have called attention only to one small portion of the great field of practical dentistry and only to a single feature of that field, but I consider it one of the most important notwithstanding it is so commonplace. Its proper mastery will sometimes tax all our energies and ingenuity and we shall grow in perfection by striving constantly to excell. If we are not more capable in our work this year than last, we have lost. As the good soil, however rich it may be, cannot be productive without strenuous culture, so the mind and hand without cultivation cannot acquire the more skillful and higher technique demanded in dental surgery. The man who is a live wire in the profession is the one whose opportunities are so great that his responsibilities become a pleasure rather than a burden,—for our profession has reached that stage of development when it is no longer the one who knows everything, but the one who knows how to look for everything and strives with all his powers to overcome obstacles and succeeds in accumulating skill as well as knowledge.

And after all is said and done in the long battle through life, it's nothing against you to fall down flat, but to stay there is a disgrace. "The harder you are thrown the higher you will bounce." Be proud of your blackened eye. It isn't the fact that you're licked that counts, but how did you fight, and why?

